# Vaccination versus SARS-CoV-2 Omicron: three vaccine doses win the battle

**DOI:** 10.1038/s41392-022-01000-3

**Published:** 2022-04-27

**Authors:** Grzegorz Maria Popowicz, Krzysztof Pyrc, Kamyar Hadian

**Affiliations:** 1 Helmholtz Zentrum München, German Research Center for Environmental Health (GmbH), Institute of Structural Biology, Ingolstädter Landstrasse 1, 85764 Neuherberg, Germany; 2 Virogenetics Laboratory of Virology, Malopolska Centre of Biotechnology, Jagiellonian University, Gronostajowa 7a, 30-387 Krakow, Poland; 3 Helmholtz Zentrum München, German Research Center for Environmental Health (GmbH), Research Group Cell Signaling and Chemical Biology, Institute of Molecular Toxicology and Pharmacology, Ingolstädter Landstrasse 1, 85764 Neuherberg, Germany

**Keywords:** Infectious diseases, Vaccines

The here highlighted study by Garcia-Beltran et al. demonstrates that SARS-CoV-2 Omicron can largely escape the immune responses. Hence, while two doses of COVID-19 vaccines seem to be insufficient to provide protection against Omicron, a third dose provides mature neutralizing antibodies to enhance the response.^1^

Since March 2020, when the World Health Organization (WHO; www.who.int) officially declared the COVID-19 pandemic caused by the SARS-CoV-2, life has been put upside down. COVID-19 has caused millions of deaths worldwide and immensely hit the global economy, social life, and people’s mental health. Intriguingly, scientists have quickly developed effective vaccines. All vaccines approved in the EU and the US contain either nucleic acids coding for the spike (S) protein or the spike protein itself. The S protein is the major surface protein of SARS-CoV-2, which together with the envelope and membrane proteins forms the virion sphere, encapsulating the genomic RNA. The S protein binds to cell surface molecules and facilitates viral entry. For this reason, the S protein has been the most common target for neutralizing antibodies. Importantly, S-based vaccines also induce T-cell mediated immunity. Clinical trial results on the vaccine efficacy yielded stunning results, showing almost complete protection from severe disease and death. However, the protection of a two-vaccine-dose regimen started waning over time, hence, demanding additional vaccine doses.

Coronaviruses are RNA viruses and thus undergo constant evolution resulting in new genetic variants (e.g., Alpha, Beta, Gamma, and Delta), of which some became globally dominant. November 2021 has seen the emergence of the new variant Omicron (B.1.1.529/BA.1). This variant was initially discovered in Botswana and South Africa, which became globally dominant in about 2 months. Omicron comprises a vast number of mutations, with more than 30 non-synonymous ones in the S protein.^1^ This immediately raised concerns that this variant may elude the immune protection induced by vaccines or the disease itself. Thus, it has been imperative to demonstrate the efficacy of vaccination against SARS-CoV-2 variants—especially Omicron—and reason for the third (booster) dose administrations.

Garcia-Beltran et al.^1^ demonstrate that SARS-CoV-2 Omicron can escape the antibody-mediated neutralization developed in response to prior vaccination or infection. Omicron is a result of bidirectional evolution, where ACE2-dependent infectivity is significantly increased (~2-fold compared to Delta), and the Omicron spike protein is able to escape neutralizing antibodies. This study utilized sera collected from community and healthcare workers with different immunization history—(1) two vaccine doses; (2) two vaccine doses + infection; (3) two vaccine doses + booster shot—and compared their neutralizing activity. Notably, the authors also considered whether the individuals were recently (<3 months) or distantly (6–12 months) vaccinated. The data demonstrate that two doses of COVID-19 vaccines were insufficiently neutralizing Omicron. This observation was similar in recently or distantly double-vaccinated individuals. Only after an additional, third booster dose or an additional infection following the vaccination, sufficient levels of cross-reactive and neutralizing antibodies were produced. The third dose of mRNA-1273 elevated neutralizing capacity by 19-fold, and the third dose of BNT162b by 27-fold. Notably, boosting the immune system with a third spike exposure was more effective upon mRNA vaccination compared to a virus infection. Together, this study reveals that three doses of the vaccine should be recommended to retain protection against SARS-CoV-2 Omicron.^1^

Other researchers also investigated the ability of two versus three vaccine shots to neutralize Omicron. Two of these studies have been carried out by Carreño et al.^2^ and Wratil et al.^3^. In the study by Carreño et al., the authors analyzed sera from (1) convalescent, (2) mRNA double vaccinated, (3) mRNA triple vaccinated, (4) convalescent plus double vaccinated, and (5) convalescent plus triple vaccinated individuals, and investigated neutralization against Wildtype, Delta and Omicron. This study concluded that convalescent or double vaccinated sera have close to undetectable neutralizing activity against Omicron. All other combinations that had exposed the immune system to the spike antigen three or four times displayed sufficient neutralizing capacity against Omicron; however, this was less pronounced when compared to Wildtype or Delta.^2^ Similarly, the study by Wratil et al. investigated neutralizing activity of sera from (1) convalescent and (2) infection-naive individuals prior to vaccination and after first, second, and third BNT162b2 mRNA vaccination. Convalescent or infection-naive individuals with two vaccinations showed good neutralization activity against Alpha, Beta, and Delta. However, while sera from convalescent individuals plus two vaccinations (three exposures) neutralized Omicron, infection-naive individuals with two vaccinations (two exposures) barely neutralized Omicron. Only a third vaccination elevated antibody levels to neutralize Omicron. Hence, also this study demonstrates that three exposures (3x vaccination; or 2x vaccination + 1x infection) are necessary to gain sufficient neutralization activity against Omicron.^3^

**Fig. 1:**
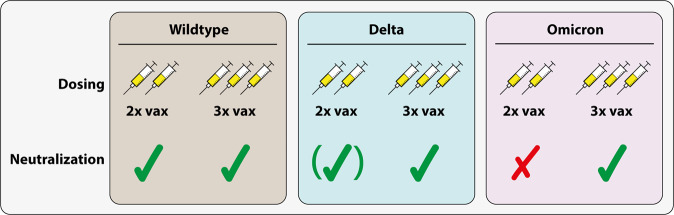
Two vaccine doses are not capable of neutralizing SARS-CoV-2 Omicron. Only three doses generate enough cross-reactive antibodies to neutralize Omicron

Together, the here highlighted study by Garcia-Beltran et al.^1^ in agreement with the studies by Carreño et al.^2^ and Wratil et al.^3^ demonstrate that the immune system has to see the spike protein at least three times in order to generate mature neutralizing antibodies with high affinity and avidity to hamper Omicron (Fig. 1). Interestingly, while an infection plus two vaccinations is enough to neutralize Omicron, three rounds of vaccination may even be better. Based on these studies, there is a clear recommendation for booster vaccination to prepare the immune system to effectively fight Omicron. One limitation of these studies is the relatively short period of observation after the booster dose. It needs to be seen whether neutralizing antibody levels after the third dose may wane over time.

In addition to the necessary three-dose vaccination regimen, drugs will be important to cope with the immune escape of Omicron. Alongside the RNA polymerase inhibitors remdesivir^TM^ and molnupiravir^TM^, other viral enzymes have been subject to drug development. Especially the viral proteases (M^pro^ and PL^pro^) are encouraging drug targets. Paxlovid^TM^ is a recently approved drug to inhibit M^pro^ activity.^4^ PL^pro^ inhibitors are still under preclinical development with acriflavine^5^ being the most potent. Excitingly, viral protease inhibitors have the potential to provide pan-betacoronavirus activity.

In conclusion, triple-vaccination together with antiviral drugs should provide adequate protection against SARS-CoV-2 Omicron and potential future variants until polyvalent vaccines are developed.
